# Modulation of Innate Immune Signaling Pathways by Herpesviruses

**DOI:** 10.3390/v11060572

**Published:** 2019-06-21

**Authors:** Qizhi Liu, Youliang Rao, Mao Tian, Shu Zhang, Pinghui Feng

**Affiliations:** Section of Infection and Immunity, Herman Ostrow School of Dentistry, Norris Comprehensive Cancer Center, University of Southern California, 925 W 34th Street, Los Angeles, CA 90089, USA; qizhiliu@usc.edu (Q.L.); youlianr@usc.edu (Y.R.); maot@usc.edu (M.T.); zhan799@usc.edu (S.Z.)

**Keywords:** innate immunity, IFN-independent ISGs, herpesvrial modulation

## Abstract

Herpesviruses can be detected by pattern recognition receptors (PRRs), which then activate downstream adaptors, kinases and transcription factors (TFs) to induce the expression of interferons (IFNs) and inflammatory cytokines. IFNs further activate the Janus kinase-signal transducer and activator of transcription (JAK-STAT) pathway, inducing the expression of interferon-stimulated genes (ISGs). These signaling events constitute host innate immunity to defeat herpesvirus infection and replication. A hallmark of all herpesviruses is their ability to establish persistent infection in the presence of active immune response. To achieve this, herpesviruses have evolved multiple strategies to suppress or exploit host innate immune signaling pathways to facilitate their infection. This review summarizes the key host innate immune components and their regulation by herpesviruses during infection. Also we highlight unanswered questions and research gaps for future perspectives.

## 1. Introduction

The innate immune response serves as the first line of defense against viral infections. Pattern recognition receptors (PRRs) sense pathogen-associated molecular patterns (PAMPs) to distinguish self from non-self. To date, Toll-like receptors, cytosolic sensors, and to a lesser extent, C-type lectins have been characterized as PRRs in mammalian cells. When activated by cognate PAMPs, PRRs dimerize with their corresponding adaptors to induce the activation of IKKα/β and TBK-1/IKKε kinase complexes. The exception is cyclic GMP-AMP synthase (cGAS) that catalyzes the synthesis of cyclic GMP–AMP (cGAMP), which in turn serves as a second messenger to activate the endoplasmic reticulum (ER)-anchored stimulator of interferon genes (STING). These two activated kinases promote nuclear factor kappa-light-chain-enhancer of activated B cells (NF-κB) and interferon regulatory factor (IRF) activation, leading to the up-regulated expression and subsequent production of inflammatory cytokines such as IFNs. When bound to IFN receptors, IFNs further activate the Janus kinase-signal transducer and activator of transcription (JAK-STAT) pathway, inducing the expression of hundreds of genes, known as IFN-stimulated genes (ISGs), to establish an antiviral state [1].

**Figure 1:**
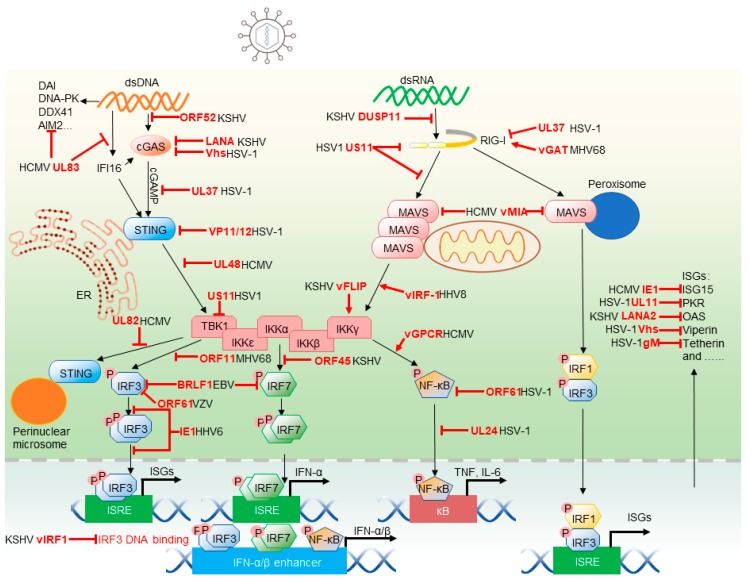
Herpesviruses modulate cytosolic pattern recognition receptors (PRR)-mediated innate immune signaling pathways. Upon herpesvirus infection, retinoic acid–inducible gene 1 (RIG-I) and cyclic GMP-AMP (cGAMP) synthase (cGAS) or other DNA sensors spot viral dsRNA and dsDNA, respectively. RIG-I initiates mitochondrial antiviral-signaling protein (MAVS) aggregation to drive signaling transduction, while cGAS recruits stimulator of interferon genes (STING) along with cyclic GMP–AMP (cGAMP) to activate kinase for later transcription activities. Phosphorylated IRF3 dimer, IRF7 dimer, IR1/IRF3 or activated NF-κB translocate to the nucleus, where they transactivate corresponding promoters to induce the expression of interferons (IFNs) and interferon-stimulated genes (ISGs). Multiple steps in cytosolic PRR-mediated innate immune signaling pathways are inhibited or hijacked by herpesviruses. An example for each escape mechanism is given. The blind-ended symbols and arrows indicate the inhibition and hijacking by herpesvirus proteins, respectively.

Herpesviruses, enveloped viruses containing double-stranded genomes, are prevalent in nature and establish life-long persistent infections. Human herpesviruses are classified into three sub-families: α-herpesvirinae, including herpes simplex virus 1 (HSV-1), 2 (HSV-2), and varicella-zoster virus (VZV); β-herpesvirinae, composed of human cytomegalovirus (HCMV), human herpesvirus 6 (HSV-6), and 7 (HSV-7); and γ-herpesvirinae, including Kaposi’s sarcoma-associated herpesvirus (KSHV) and Epstein–Barr virus (EBV) [2]. After viral entry, the transcription, herpesviral genome replication, and capsid assembly of lytic replication occur in the host cell nucleus of herpesvirus-infected cells. Viral genes are categorized into three temporal classes that demonstrate sequential expression: (1) immediate-early genes encoding regulatory proteins, (2) early genes encoding enzymes for replicating viral DNA, and (3) late genes encoding structural proteins [3]. The tegument and envelope are generated and assembled upon virion budding at the trans-Golgi network (TGN) or plasma membrane. Virions are transported to the cell membrane by TGN vesicles and released as mature virions via fusion with the plasma membrane. Alternatively, viruses undergo a latent state, which can be reactivated to lytic replication by environmental cues such as stress and immunosuppression [4]. To evade host innate immune responses, herpesviruses have evolved multiple strategies to inhibit and hijack key signaling components. Though numerous innate immune responses against herpesvirus infection and viral replication have been extensively studied, we will emphasize recent advances on the interaction between herpesvirus and the key molecules in innate immunity (Figure 1).

## 2. Modulation of Inflammatory Response by Herpesviruses

### 2.1. Cytosolic Sensors and Adaptors

Central to innate immune activation is the pattern recognition receptor that senses PAMPs. Along with membrane-anchored Toll-like receptors (TLRs) and lectins that monitor extracellular compartments and intracellular vesicles, cytosolic sensors patrol the cytosol for foreign or aberrantly localized molecular signatures or PAMPs. Cytosolic receptors relevant to herpesvirus infection are the retinoic acid–inducible gene 1 (RIG-I), cGAS and Interferon Gamma Inducible Protein 16 (IFI16) sensors that detect dsRNA and dsDNA. The role of melanoma differentiation-associated protein 5 (MDA5) in herpesvirus infection is not well defined, although it was previously reported that human primary macrophages can recognize HSV-1 via MDA5 [5] and that MDA5 exerts antiviral effect on KSHV lytic replication [6]. Upon association with cognate ligands, these cytosolic sensors signal to activate NF-κB and IRF transcription factors (TFs) that, in turn, promote the expression of inflammation-associated genes.

TLRs recruit their adaptor molecules, such as myeloid differentiation primary response 88 (MYD88) and TIR-domain-containing adapter-inducing interferon-β (TRIF) to the plasma membrane. Adaptor molecules of the cytosolic sensors anchor their corresponding signalosomes to membranes of distinct intracellular compartments. Mitochondrial antiviral-signaling protein (MAVS) and STING localize to the mitochondrion, and ER, respectively. Interestingly, MAVS can reside in the mitochondrion-associated membrane (MAM) and peroxisomes [7,8]. In doing so, these adaptor molecules enable their organelle-specific activation that is likely connected to the physiological function of individual subcellular compartments. Herpesviruses dedicate various factors to manipulate these adaptor molecules to attenuate cellular signal transduction at their discretionary disposition. 

#### 2.1.1. RIG-I-MAVS 

A prototype member of the RIG-I-like receptor (RLR) family, RIG-I consists of two N-terminal caspase recruitment domains (CARDs), a central DEAD box helicase/ATPase domain and a C-terminal regulatory domain (CTD) [9,10]. Via the CTD, RIG-I recognizes short dsRNAs containing a 5 di-phosphorylate or tri-phosphorylate moiety that marks viral replication intermediates [11,12]. When RIG-I binds dsRNA, the CTD and the helicase domains form an “o” ring-like structure that wraps around the dsRNA helix, unleashing the N-terminal CARDs from auto-inhibition to dimerize with the CARD of MAVS. Unlike RIG-I, the MDA5 CARDs are not restrained by other domains in the absence of dsRNA. However, when binding to viral dsRNA, MDA5 wraps around the dsRNA in a similar way and undergoes conformational change, resulting in clustering of CARD domains. Consequentially, the oligomerized MDA5 serves as the scaffold to interact with MAVS. MAVS resides in the outer membrane of the mitochondrion and, through CARD-mediated oligomerization, heterodimerizes with RIG-I and homodimerizes to form prion-like fibers [13]. CARD-mediated intramolecular interaction and other structural motifs for recruiting key factors such as TRAFs and immune kinases are central for the MAVS-mediated signaling [14,15,16]. MAVS is also located at peroxisomes that mediate a rapid IFN-independent expression of inflammatory genes, which contributes to the antiviral activity of MAVS [8]. A model has been proposed in which viral dsRNA could be detected by RLRs to activate peroxisome-localized MAVS and induce the immediate expression of ISGs, but not IFNs. These experiments were performed in mouse embryonic fibroblast (MEF) cells, while cells deficient in IRF1 or IRF3 were not able to induce ISG expression via peroxisome-localized MAVS [8]. However, Bender et al. found that IFN-α, IFN-β, IFN-λ1–3 can be induced by both mitochondrial and peroxisomal MAVS in A549 cells [17]. The peroxisomal MAVS-mediated signaling pathway engenders an immediate and transient antiviral effect, which enables cells to express ISGs. Nevertheless, it is still controversial whether peroxisomal MAVS is essential for IFN induction.

It is counter-intuitive that RIG-I is important for herpesvirus infection, given that herpesviruses contain DNA genomes. Accumulated studies demonstrate that RIG-I can sense RNA produced from cells infected by various herpesviruses, including herpes simplex viruses (HSV) [18], Kaposi’s sarcoma-associated herpesvirus (KSHV) [6], murine gamma herpesvirus 68 (MHV68) [19] and Epstein–Barr virus (EBV) [20]. Subsequent studies hunting for RIG-I ligands identified diverse cellular and viral RNAs that are sufficient to induce RIG-I-mediated signaling. These ligands include cellular RNAs derived from a ribosomal RNA pseudogene in HSV-1-infected cells or aberrant spliced cellular mRNAs and viral RNA transcripts in KSHV-infected cells [6,21,22]. Surprisingly, the primary species pulled down by RIG-I were mutually exclusive in two independent studies using KSHV-infected cells [6,22]. Although the reason underlying the discrepancy remains unknown, this may stem from the distinct cell lines and experimental conditions used for their studies. The observations that cellular noncoding and aberrantly processed dsRNA are recognized by RIG-I suggest the physiological function of RIG-I beyond innate immune defense against microbial infection [6].

To circumvent RIG-I-mediated antiviral immunity, herpesviruses deploy two general strategies: either interfering with the RNA-binding of RIG-I or degrading/modifying dsRNA. Notably, gamma herpesviruses, such as KSHV, EBV and MHV68, encode one or several viral homologues of cellular glutamine amidotransferases, hence referred to as vGAT. Cellular glutamine amidotransferases are responsible for the synthesis of nucleotides, amino acids, glycoproteins and an enzyme cofactor (NAD). vGAT recruits a cellular glutamine amidotransferase to induce RIG-I deamidation. In fact, vGAT shares low, but significant, homology with cellular phosphoribosylformylglycinamidine synthetase (PFAS), which catalyzes the fourth step of the de novo purine synthesis pathway. Deamidated RIG-I is activated without dsRNA to induce IKKβ kinase activity that is usurped to promote viral replication in the case of MHV68 infection [23]. Importantly, vGAT-induced deamidation renders RIG-I unable to sense dsRNA, a mechanism that is shared by HSV-1 UL37. In contrast to gamma herpesvirus vGAT proteins, HSV-1 UL37 is a bona fide deamidase that is sufficient to deamidate RIG-I in vitro and in cells [18]. These studies re-discover the regulatory role of protein deamidation in regulating innate immune sensing of nucleic acids by RIG-I.

The virion host shutoff (Vhs) is a bona fide RNase that degrades dsRNA [24,25] to minimize RNA detection by RIG-I, and likely other RNA sensors such as dsRNA-dependent protein kinase R (PKR) and MDA5. RIG-I recognizes the stem region and the 5’-end phosphate group of a di- or tri-phosphate moiety [26]. Supporting the crucial role of the 5’ di- or triphosphate appendage, KSHV was shown to induce DUSP11, a dual specificity phosphatase, to dephosphorylate 5’-phosphate-containing dsRNA, thus reducing RIG-I-mediated innate immune activation [6]. When RNA-binding of RIG-I is concerned, viral or cellular RNA-binding proteins can be mobilized to sequestrate dsRNA and avoid RIG-I activation. Alternatively, proteins encoded by herpesviruses (such as HSV-1 US11) physically associate with RIG-I to either exclude RNA-binding or interfere with RIG-I oligomerization or hetero-dimerization with the MAVS adaptor [27]. Collectively, these studies elucidate diverse viral immune evasion strategies and hint how RIG-I, a pivotal cytosolic sensor, is regulated under high scrutiny during herpesvirus infection.

HCMV has shown to promote fragmentation of mitochondrion by viral mitochondrion-localized inhibitor of apoptosis (vMIA), thus dampening signaling downstream of MAVS [28]. HCMV vMIA has also shown to directly interact with peroxisomal MAVS and inhibit the antiviral signaling pathway downstream of peroxisomal MAVS [29]. However, unlike the mitochondrial MAVS, vMIA-induced fragmentation of peroxisomes did not explain the inhibition of antiviral signaling, suggesting an unknown mechanism [29]. Herpesviruses also target these two organelles to derail MAVS-dependent innate immune activation, suggesting a potential linkage between intracellular organelles and innate immune response [30,31,32]. 

#### 2.1.2. cGAS-STING

In eukaryotic cells, cGAS plays a pivotal role in sensing dsDNA in the cytosol [33,34]. Structural studies indicate that the C-terminal Mab21 domain binds dsDNA to induce dimerization and activation of its cGAMP synthetase activity [35]. Recently, the long N-terminal domain was shown to regulate DNA-binding via phase separation that can be modulated by post-translational modifications such as phosphorylation [36,37]. Other post-translational modifications, including glutamylation [38], sumoylation [39] and acetylation [40] modulate the activation and/or enzyme activity of cGAS, thus fine-tuning the innate immune activation downstream of cGAS. Activated cGAS catalyzes the synthesis of cGAMP that serves as a second messenger. Anchoring in the endoplasmic reticulum (ER), STING binds cGAMP and undergoes dimerization and higher-order oligomerization [41]. Oligomerized STING translocates to the trans-Golgi network (TGN) [42], where the addition of K27-linked [43] and K63-linked poly-ubiquitin chains to STING are catalyzed by distinct E3 ubiquitin ligases [44,45]. Serving as an anchor, K27-linked and K63-linked poly-ubiquitin chains recruit TBK1, which in turn phosphorylates STING. TBK1-mediated phosphorylation endows STING with a negatively charged surface that recruits IRF3 via a positively charged surface. As such, IRF3 is efficiently phosphorylated by TBK1 due to close proximity [14]. STING also recruits TBK1 and TRAF6 to activate NF-κB, which promotes inflammatory cytokine production. Finally, STING translocates to the perinuclear microsomes, where it undergoes lysosome-dependent degradation, thereby terminating STING-mediated innate immune activation [46,47]. 

Despite the herpesvirus DNA genome being encapsidated while it isdelivered into the nucleus, viral genomes can leak out from nucleocapsids and are recognized by cGAS in the cytosol [48]. However, recent studies also suggest the possible nuclear role of cGAS [49] and RIG-I [50] in innate immune detection. Supporting the pivotal role of cGAS in host defense against HSV-1 infection, mice deficient in cGAS and its downstream adaptor STING were more susceptible to HSV-1 than wild-type mice [34]. HCMV-infected monocyte-derived cells generated abundant cGAMP, and knockdown or knockout of cGAS in THP-1 monocytes and primary monocyte-derived cells attenuated IFN-I response, supporting the conclusion that cGAS senses HCMV infection [51].

Herpesviruses have evolved distinct strategies to avoid cGAS activation. This is achieved via interfering with DNA-binding and/or cGAMP-synthase activity of cGAS. KSHV encodes a small tegument protein, ORF52 (named as KSHV inhibitor of cGAS, kicGAS) that binds dsDNA to prevent the DNA-binding and activation of cGAS [52]. Homologues of ORF52 encoded by EBV, rhesus monkey rhadinovirus (RRV) and MHV68 appear to possess similar inhibitory effect on cGAS, despite the amino acid identity being low among these viral cGAS inhibitors [53,54,55,56]. KSHV also expresses a short form of the nuclear latency-associated antigen (LANA) that localizes in the cytoplasm [57]. This isoform of LANA was shown to bind cGAS and inhibit its cGAMP production [58]. Human cytomegalovirus UL31 binds to cGAS and dissociates cGAS-dsDNA complex, thus reducing the enzymatic activity of cGAS [59]. Similarly, UL83 (also known as pp65) of HCMV interacts with cGAS and attenuates cGAS activation [60]. Interestingly, cGAS was found to colocalize in the nucleus with UL83, supporting the potential role of cGAS in the nucleus [60]. cGAS was also demonstrated to modulate IFI16-dependent antiviral activity in the nucleus, although the nuclear function of cGAS remains elusive [61,62]. The UL37 deamidase of HSV-1 targets cGAS, in addition to RIG-I, for deamidation [63]. While deamidation does not reduce the DNA-binding and dimerization of cGAS, it specifically ablates the cGAMP synthase activity of cGAS. Moreover, most nonhuman primate cGAS proteins contain natural variations at the site proximal to the catalytic triad of cGAS which renders nonhuman primate cGAS proteins resistant to UL37-mediated deamidation and HSV-1 evasion [63]. HSV-1 Vhs protein downregulates cGAS expression via accelerated mRNA degradation to inhibit cGAS-mediated signaling, providing an example of immune advantage due to “collateral damage” of the RNase activity of Vhs [64]. Taken together, herpesviruses evolved diverse intricate strategies to counteract the cGAS sensor during infection. 

Herpesviruses target multiple steps intrinsic to STING activation to attenuate innate immune response downstream of cGAS. VP11/12 encoded by HSV-1 and IE86 protein encoded by HCMV trigger the degradation of STING to suppress STING-dependent innate immune defense [65,66]. The molecular action of these herpesvirus proteins remains unknown, although it is proposed that IE86 degrades STING in a proteasome-dependent manner. HCMV UL82 blocks the translocation of STING to the peri-nuclear microsomes by disrupting the STING-iRhom2-TRAPβ translocation complex, and impairs the recruitment of TBK1 and IRF3 to the STING signalosome. In doing so, it identifies key components, iRhom2 and RTAPβ, in STING subcellular translocation and signaling [67]. HCMV UL48 and MHV68 ORF64 proteins, functioning as deubiquitinases (DUBs), deubiquitinate STING to impede the recruitment of TBK1 and downstream signaling events of the STING signalosome [68,69]. These findings highlight the organelle-specific translocation of STING and regulation thereof by herpesvirus in innate immune signaling. 

#### 2.1.3. IFI16

IFI16, a member of the PYHIN protein family, is an innate immune sensor for detecting intracellular DNA. IFI16 contains a pyrin domain that mediates inflammasome activation, and two DNA-binding HIN domains that detect viral dsDNA (such as HSV 60mer). IFI16 primarily localizes in the nucleus, but is also found in the cytoplasm and mitochondria at detectable levels [70,71]. Upon DNA transfection or HSV-1 infection, IFI16 is required for the activation of IRF3 and NF-κB [71]. During HCMV infection, activated IFI16 translocates into the cytoplasm and binds to HCMV DNA [72]. Additionally, IFI16 interacts with cGAS via its pyrin domain, which stabilizes cGAS to promote its protein expression [73,74]. Overexpression of IFI16 enhances the cGAS activity, while depletion of IFI16 reduces cGAS expression and impairs cGAMP production in macrophages [61]. Further, IFI16 promotes STING phosphorylation and translocation, resulting in elevated activation of STING induced by cGAMP [62]. To evade host innate immune responses, HCMV tegument protein UL83 blocks DNA sensing of IFI16 by interacting with the pyrin domain to prevent the formation of a nuclear oligomer [75]. HSV-1 ICP0 was shown to degrade IFI16 via its E3 ligase activity [76], although this result was not supported by another study [77]. Roy et al. found that IFI16 is polyubiquitinated and degraded in a proteasome-dependent manner in tissue plasminogen activator (TPA)- or doxycycline-induced cells expressing lytic KSHV proteins [78], suggesting that KSHV gene products degrade IFI16 upon lytic reactivation. These findings collectively show that IFI16 is critical for host defense against herpesvirus infection.

#### 2.1.4. Other Herpesvirus Sensors

In addition to the three main nucleic acid sensors described above, other PRRs were investigated in the context of herpesvirus infection. IFN-inducible protein absent in melanoma 2 (AIM2) detects cytosolic DNA [79]. The HIN200 domain of AIM2 binds to DNA, whereas the pyrin domain interacts with the adaptor molecule, an apoptosis-associated speck-like protein containing a caspase activation and recruitment domain (ASC). Activated AIM2 dimerizes with ASC to form large speckles in the cytosol that provoke the activation of NF-κB and caspase-1 [80]. The AIM2 inflammasome can be activated by herpesviruses, such as mouse CMV [81]. The important role of the AIM2 inflammasome in herpesvirus infection is reflected by inhibition displayed by several herpesviruses. For example, HCMV UL83 interacts with AIM2 and inhibits its inflammasome activity [82]. HSV-1 VP22 disrupts AIM2 oligomerization via a physical interaction, thus inhibiting the AIM2 inflammasome to promote viral replication [83]. Additionally, DNA-dependent activator of IFN-regulatory factors (DAI), DNA-dependent protein kinase (DNA-PK) and DEAD box helicase 41 (DDX41) are implicated in sensing herpesvirus DNA, but their exact functions in herpesvirus infection are not well understood [84,85,86]. TLRs also recognize invading herpesvirus located in their extracellular compartments or intracellular vesicles. TLR2 and TLR4 sense virion components; TLR3 and TLR7 detect viral RNA; and TLR9 recognizes viral genomic DNA [87,88,89]. Future studies examining the in vivo functions of these sensors are needed to further the mechanistic understanding of innate immune responses against herpesvirus pathogenesis. 

### 2.2. Immune Kinases and Transcscription Factors

Kinases are responsible for signal amplification via phosphorylating downstream signaling components that lead to transcriptional activation. Specifically, IKKβ and TBK1 drive the activation of NF-κB and IRF3 to up-regulate the expression of inflammatory genes, including the IFN-independent ISGs [90,91]. Both IKKβ and TBK1 are central components of the immune kinase complex that contain an additional scaffold protein, NEMO and TANK, respectively [90,92]. Herpesviral proteins modulate these two kinases via interactions with components of their corresponding complexes. 

NF-κB TFs consist of five members (i.e., RelA, RelB, c-Rel, p50 and p52). Among them, p50 and p52 are processed from their precursors, p105 and p100, respectively. NF-κB TFs regulate an array of biological processes that are relevant to viral infection [93]. Most notable cellular activities include immune response and the apoptosis/survival of virus-infected cells. The best-studied NF-κB TF in the context of viral infection is the RelA-p50 dimer, which is the prototype NF-κB. Key events of NF-κB activation consist of phosphorylation and degradation of IκB, nuclear translocation, and binding to gene promoters of RelA-p50 and recruitment of co-activator (specifically CBP/p300) and RNA polymerase II [94,95]. Herpesviruses target these steps to alter NF-κB-dependent gene expression. 

In collaboration with NF-κB and other transcription factors, IRFs up-regulate the expression and production of IFNs. Additionally, IRF TFs directly transactivate the expression of diverse ISGs to defeat invading viral pathogens, providing an immediate and intrinsic antiviral activity in response to viral infection. For example, IRF3 and IRF7 are crucial for the expression of ISGs (such as Mx and viperin) that occur concomitantly to IFN induction, thus contributing to antiviral defense preceding IFN-mediated response [1]. To inhibit IFN and ISG expression, multiple strategies have been used by herpesviruses to block IRF3/7-mediated transcription activation.

#### 2.2.1. IKKβ-NF-κB 

Herpesvirus both activate and inhibit the NF-κB pathway, mostly contributing to the survival of infected cells or evasion of triggered antiviral defense respectively. To promote the survival of the infected cells, the FLICE inhibitory protein (vFLIP) of KSHV was found to interact with the NEMO (IKKγ) subunit to activate IKKα/β and NF-κB. NF-κB activation is crucial for the survival of KSHV latently infected lymphoma cells [96,97,98]. Similarly, EBV encodes latent membrane protein 1 (LMP1) that activates NF-κB via both canonical and non-canonical pathways [99]. LMP1-induced NF-κB activation was shown to promote the survival of EBV-infected cells and enable the EBV-regulated gene expression, both of which are implicated in EBV transformation and malignancies of EBV infection [99].

Lysophosphatidic acid activates G protein-coupled receptors (GPCR), leading to the activation of protein kinase C (PKC). Accordingly, the activated PKC phosphorylates caspase recruitment domain (CARD)-containing proteins (CARMA) and associate with IKKβ to activate NF-κB [100,101]. HCMV and KSHV encode several vGPCRs, which are homologous to the human IL-8R, and also activate NF-κB through distinct mechanisms [102,103]. These vGPCRs are constitutively active, although agonists can further promote vGPCR-mediated signaling. Paradoxically, vGPCRs are expressed in the lytic phase and implicated in pathogenesis of these herpesviruses, likely via both autocrine and paracrine mechanisms [102,103]. NF-κB activation by vGPCRs appears to be less robust than that induced by vFLIP and LMP1. Along with its constitutive active nature, vGPCRs may contribute to the chronic activation of NF-κB, an inflammatory angiogenic microenvironment and malignancy associated with these viral pathogens. 

HSV-1-encoded UL36 deubiquitinates IκBα to inhibit its degradation, thereby locking NF-κB in an inactivated form and blocking NF-κB activation [104]. Similarly, the ORF61 proteins encoded by Simian Varicella Virus (SVV) and Varicella-zoster virus (VZV) prevent IκBα from ubiquitination by interfering with the ubiquitin ligase of IκBα [105]. SVV additionally prevents IκBα from phosphorylating, which is required for ubiquitination and subsequent degradation by the proteasome pathway. Both UL24 and UL42 encoded by HSV-1 bind to NF-κB subunits p65 and p50 to abolish their nuclear translocation, thus suppressing NF-κB activation [106,107]. HCMV-encoded UL26 inhibits the phosphorylation and activation of IKKβ required for IκB phosphorylation and NF-κB activation [108]. Interestingly, γHV68 usurps RIG-I and MAVS to activate IKKβ, which is directed to phosphorylate RelA at serine 468 [31]. Phosphorylated RelA is degraded by the proteasome in cells infected with MHV68, thereby shutting down antiviral cytokine production. These studies define diverse mechanisms by which NF-κB is differentially regulated during herpesvirus infection.

#### 2.2.2. TBK1-IRF3 

HSV-1 ICP27 inhibits IRF3 activation by interacting with the activated STING-TBK1 signalosome. Upon HSV-1 infection, ICP27 can co-immunoprecipitate with STING and TBK1. However, in cells deficient in either STING or TBK1, ICP27 loses interaction with both molecules, indicating that both STING and TBK1 are required for their interactions with ICP27. IRF3 phosphorylation, but not TBK1 autophosphorylation, is inhibited by ICP27, indicating that ICP27 interrupts signal relays from TBK1 to IRF3 [109]. Similarly, MHV68 ORF11 reduces the interaction between TBK1 and IRF3. A central domain of ORF11 traps the kinase domain to inhibit TBK1 binding to IRF3. Therefore, ORF11 inhibits TBK1-mediated IRF3 phosphorylation and downstream signaling events that lead to interferon production [110]. Interestingly, TBK1 was found to associate with heat shock protein Hsp90, which stabilizes TBK1 to enhance its protein expression. HSV-1-encoded US11 protein displaces TBK1 to interact with Hsp90, leading to the rapid degradation of TBK1 in a proteasome-dependent manner. Therefore, herpesviruses evolved diverse measures to interrupt signal transduction between TBK1 and IRF3 to impair ISG expression [111].

To inhibit IFN and ISG expression, herpesviruses demonstrate various means to block IRF3-mediated transcription activation. These steps include the stability, phosphorylation, nuclear translocation, DNA binding and recruitment of CBP of IRF3. VZV protein ORF61 promotes the ubiquitination of phosphorylated IRF3, thus accelerating its degradation [112]. Additionally, IE62 and ORF47 of VZV inhibit IRF3 phosphorylation with distinct specificity. While IE62 abolishes the phosphorylation of S396 and S398, ORF47 acts on S402 and S396. How these two viral proteins specifically affect peculiar residues remains anopen question [113,114]. Interestingly, human herpesvirus 6 immediate-early 1 protein (IE1) attenuates the IRF3 dimerization and nuclear translocation induced by TBK1 and IKKε [115]. KSHV vIRF1, MHV68 ORF36 and HSV UL13 all prevent IRF3 from interaction with CBP, thus impeding RNA polymerase II recruitment to promoters containing the IRF3-responsive element [116,117]. K-bZIP, a leucine zipper-containing transcription factor encoded by KSHV ORF K8, competitively binds to the PRDIII-I region of the IFN-β promoter, preventing the IRF3-depedent transcription of IFN and presumably those IFN-independent ISGs [118]. Similarly, EBV BGLF4 kinase impairs IRF3 binding to DNA by phosphorylating three sites within a proline-rich region (i.e., S123, S173 and T180) [119]. These herpesvirus proteins not only elucidate viral immune strategies, but also uncover key steps by which IRF3-mediated gene expression and cellular immune response can be regulated. 

Several herpesviral proteins target IRF7 and inhibit its phosphorylation, thereby reducing IFN-α and IFN-β production. KSHV ORF45 interacts with IRF7 to block its phosphorylation and subsequent nuclear translocation [120]. EBV BZLF1 interacts with IRF7, thereby inhibiting IRF7 binding to ISRE, but not IRF7 translocation, upon dsRNA stimulation. This modulation requires the BZLF1 activation domain, but not the DNA-binding domain [121]. Later, the same group reported BRLF1 could inhibit the transcription of both IRF3 and IRF7, which are dependent on the N- and C-terminal regions of BRLF1 and its nuclear localization signal [122].

### 2.3. ISGs with Direct Antiviral Activities

ISGs, such as ISG15, viperin, tetherin, PKR and OAS, can directly restrict viral infection. To evade host defense, herpesviruses dedicate various gene products to specifically target ISGs to suppress their function. 

#### 2.3.1. ISG15

ISG15 is a 15-kDa ubiquitin-like polypeptide that is conjugated to proteins, which is known as ISGylation. The conjugation is reversible and catalyzed by a protease USP18 or UBP43 [123]. ISGylation and unconjugated ISG15 play various roles in disrupting viral life cycles such as entry, replication, and release. ISGylation is involved in cellular processes, including DNA repair, autophagy, protein synthesis, and exosome secretion [124]. Furthermore, ISGylation prevents IRF3 from Pin1-mediated ubiquitination and degradation to boost antiviral immune response [125]. Thus, ISG15 demonstrates antiviral activity in various stages of the viral life cycle via the ISGylation of cellular and viral targets. These cellular targets participate in either viral replication processes or host immune defense. ISG15 can inhibit replication of HCMV by downregulating viral gene expression and virion release [126]. In turn, HCMV IE1 and pUL26 have shown to mitigate HCMV-induced ISG15 expression. Interestingly, pUL26 interacted with ISG15 and inhibited virus-induced ISGylation independent of its own ISGylation [126]. KSHV vIRF3 was found to interact with an ISG15 E3 ligase, HERC5, and decrease ISG15 conjugation, possibly promoting KSHV replication [127].

#### 2.3.2. Viperin

Viperin is an IRF3-inducible ISG whose expression is up-regulated in the absence of IFN, thereby serving as an important early enhancer of the innate immune system in response to viral infection [128]. However, the definitive mechanism of the antiviral activity of viperin against diverse viruses is less understood [128]. Recently, a study reported that viperin catalyzes the conversion of cytidine triphosphate (CTP) to 3ʹ-deoxy-3’,4ʹ-didehydro-CTP (ddhCTP), a chain terminator of the virus-encoded RNA-dependent RNA polymerases. This study suggests viperin likely targets viral transcription and genome replication for inhibition via a viral RNA-dependent RNA polymerase [129,130]. Moreover, IRAK1 and TRAF6 enhance the enzyme activity of viperin 10-fold, catalyzing the conversion to ddhCTP. TRAF6-mediated ubiquitination of IRAK1 requires the association of viperin with IRAK1 and TRAF6, thus reinforcing the innate immune activation downstream of TLR7 and TLR9 [131]. These studies highlight a potential metabolic activity of viperin in antagonizing viral replication. HSV-1 Vhs appears to target multiple ISGs, including viperin, via its intrinsic RNase activity. In doing so, HSV-1 Vhs inhibits the protein expression of these ISGs during HSV-1 infection. In fact, these ISGs potently inhibit Vhs-null HSV-1, but not WT HSV-1, supporting the hypothesis that Vhs counteracts the antiviral activity of these ISGs [132,133].

#### 2.3.3. Tetherin

Initially described in 2009, tetherin (also known as BST-2) has since been shown to have antiviral activity against diverse enveloped viruses [134]. Tetherin inhibits viral replication by retaining enveloped progeny virions on the surface of infected cells, impeding virion release and promoting virion internalization by endocytosis [135] and subsequent degradation [136]. Specifically, tetherin blocks HIV-1 budding by anchoring two membranes (i.e., the plasma membrane of the infected cell and the envelope of HIV-1 virions) [137]. Thus, tetherin effectively traps virions on the cell surface and prevents virion release to inhibit viral productive infection. To counteract the tetherin restriction, HSV-1 Vhs reduces tetherin protein expression via its intrinsic RNase activity, while envelope glycoprotein M (gM) antagonizes tetherin with an unknown mechanism [133,138]. KSHV K5/MIR2 ubiquinated lysines in the short amino-terminal domain of thetherin, promoting its lysosomal degradation [139].

#### 2.3.4. PKR 

The dsRNA-dependent protein kinase R (PKR) is a member of the protein kinase superfamily. PKR contains a dsRNA-binding domain within the N-terminus [140,141] and a catalytic domain responsible for kinase activity within the C-terminus [142]. In the presence of ATP, dsRNA binds to PKR and triggers a conformational change that leads to PKR autophosphorylation and activation [143]. The activated PKR specifically phosphorylates the α subunit of the translation initiation factor eIF2, hence inhibiting cellular and viral protein synthesis [144]. Interestingly, in addition to eIF2, PKR phosphorylates and activates the IκB kinase, resulting in IκBα degradation and subsequent activation of NF-κB [145]. Thus, PKR antagonizes viral replication via inhibiting translation initiation and activating NF-κB. HSV1 UL11 interacts with PKR and inhibits its activation in response to dsRNA [146]. Interestingly, EBV nuclear protein BS-MLF1 (SM), homologous to the carboxyl-terminal domain of US11, also interacts with PKR and prevents PKR activation [147]. KSHV LANA2 counteracts PKR by disrupting the PKR-induced phosphorylation of eIF-2α and PKR-mediated inhibition of protein synthesis [148].

#### 2.3.5. OAS

Although 2′–5′-oligoadenylate synthetase (OAS) is abundantly expressed in IFN-treated cells, it can be expressed in an IFN-independent manner [149,150]. In the presence of ATP and dsRNA, OAS synthesizes 2′–5′ linked oligomers of adenosine (2–5 (A)) [151] that trigger the binding of RNase L to the oligomerized 2–5 (A)s and subsequently dimerize. As such, RNase L cleaves cellular and viral RNAs [152]. The RNase L-mediated RNA cleavage either directly inhibits viral translation and replication [153,154] or generates fragmented RNA to stimulate RIG-I-dependent innate immune activation [155]. Surprisingly, a recent study suggests a mouse OAS homologue (specifically, OASL1) inhibits cGAS-mediated antiviral immune response [156]. Moreover, a previous report showed that OAS1 is counter-selected in nonhuman primate species such as gorillas, probably to balance the deleterious and antiviral effects of the 2′–5′ oligo (A) synthesis [157]. The in vivo roles of OAS await further investigation. Nevertheless, OAS proteins appear to play distinct immune-modulating roles depending on the physiological context. HSV-1 US11 is sufficient to inhibit OAS activation [158]. Infection of HSV-1 in IFN-induced cells compromises the RNA cleavage activity of OAS. Inhibition of OAS requires the dsRNA-binding domain of US11, indicating that US11 may sequester available dsRNA produced during infection to prevent RNA processing mediated by OAS [158].

### 2.4. JAK-STAT Pathway

**Figure 2:**
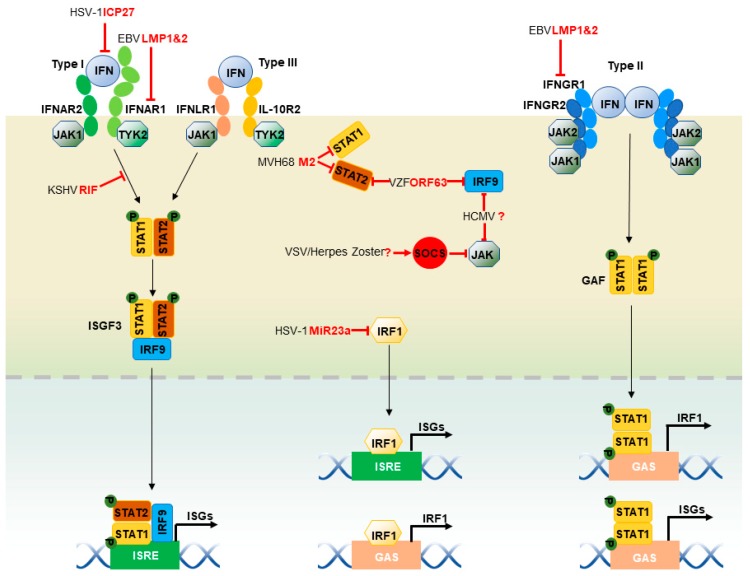
Herpesviruses modulate the JAK-STAT pathway. When IFNs are induced, Type I, II and III IFNs bind to their cognate receptor complexes. When bound to type I IFNs, IFNAR1 and IFNAR2 heterodimers recruit JAK1 and TYK2 to their cytoplasmic tails. Type III IFNs bind to IL-10R2 and IFNLR1 heterodimers to recruit JAK1 and TYK2. JAK1 and TYK2 are placed in proximity to IFN receptors and autophosphorylate to promote their activation. Activated JAK1 and TYK2 phosphorylate tyrosine residues within the intracellular carboxyl termini of STAT1 and STAT2. These phosphorylated STAT1 and STAT2 form a heterodimer that binds to IRF9, constituting the ISGF3 complex. In contrast, type II IFNs activate JAK1 and JAK2 by IFNGR1 and IFNGR2 heterodimers, leading to the phosphorylation of both JAK1 and JAK2, and the subsequent phosphorylation of receptor chains. Phosphorylated STAT1 forms a homodimer, known as GAF, and translocates into the nucleus. In addition, IRF1 can directly translocate into the nucleus and transactivate ISGs, including itself. The red lines delineate the impact of viral infection on the JAK-STAT pathway. Multiple steps of the JAK-STAT pathway are inhibited or hijacked by herpesviruses. The blind-ended lines and arrows indicate the cellular components that are inhibited and hijacked by herpesviruses, respectively. The question mark denotes an unknown herpesviral protein.

Interferons (IFNs) signal through the JAK-STAT pathway to induce antiviral gene expression (Figure 2). Type I (α and β), II (γ) and III (λ) IFNs bind to their cognate receptor complexes that often constitute a heterodimer. Both type I and type III IFNs signal by activating the Janus kinase 1 (JAK1) and the tyrosine kinase 2 (TYK2): type I IFNs act through heterodimers consisting of IFN-α receptor 1 (IFNAR1) and 2 (IFNAR2), whereas type III IFNs act through heterodimers composed of interleukin-10 receptor 2 (IL-10R2) and IFN-λ receptor 1 (IFNLR1). IFN-induced oligomerization of receptors results in the recruitment of JAK1 and TYK2, which auto-phosphorylates and further phosphorylates tyrosine residues within the intracellular carboxyl termini of the receptors [1]. Consequently, STAT1 and STAT2 are recruited to the IFN receptor complexes and phosphorylated. Phosphorylated STAT1 and STAT2 form a heterodimer that subsequently binds to IFN regulatory factor 9 (IRF9), constituting the ISG factor 3 (ISGF3) complex. In contrast, type II IFNs activate JAK1 and JAK2 through IFN-γ receptors 1 (IFNGR1) and 2 (IFNGR2) heterodimers, leading to the phosphorylation of both JAK1 and JAK2, and subsequent phosphorylation of receptor chains. STAT1 is phosphorylated by JAK1 and JAK2, which results in STAT1 homo-dimer known as the IFN-γ activation factor (GAF). Both ISGF3 and GAF translocate into the nucleus and bind to ISRE and GAS, respectively, to activate the transcription of ISGs [1]. Herpesviruses demonstrate different strategies to inhibit the JAK-STAT pathway stepwise, including the expression of IFN receptors, binding with receptors, the phosphorylation of JAK and STATs and the expression of STATs and IRF9 [159,160]. EBV-latent membrane protein LMP2A and LMP2B mediate degradation of IFNAR1 and IFNGR1 in an unknown mechanism [161]. Similarly, MHV68 M2 protein downregulates STAT1 and STAT2, resulting in inhibition of the IFN-mediated transcriptional activation [162]. EBV immediate-early protein, BZLF1, decreases expression of the IFNGR1 receptor, coupling with inhibition of STAT1 tyrosine phosphorylation and nuclear translocation upon induction [163]. HSV-1 ICP27 induces the secretion of type I IFN antagonizing protein, which competes with type I IFN receptors to bind IFNs, leading to impaired IFN signaling [164]. KSHV (ORF10) (named RIF) interacts with JAK1, TYK2, and STAT2 to form an inhibitory complex of a type I IFN signal, thus attenuating JAK1 and TYK2 activities, phosphorylation of both STAT2 and STAT1, and formation of the ISGF3 transcriptional cofactor [165]. VZV and herpes zoster up-regulates cellular IFN-stimulated suppressor of cytokine signaling (SOCS) proteins, JAK kinase inhibitors, to inhibit the JAK-STAT pathway [166], although HCMV reduces IRF9 and JAK1 expression, with no viral factors identified in these capacities [167]. VZV ORF63 inhibits IRF9 expression and STAT2 phosphorylation [168], although the mechanisms remain unknown.

Similar to IRF3 and IRF7, IRF1 binds to ISREs within ISG promoters and regulate the expression of ISGs [169,170]. IRF1 also binds to a single interferon GAS element (TTTCCCCGAAA) for regulation. Interestingly, the IRF1 promoter contains a GAS element that allows IRF1 to up-regulate its own expression, constituting a positive amplification loop [171]. Requiring no modification, IRF1 can enter the nucleus via its NLS located within the C-terminus of the DNA-binding domain [172]. IRF1 expression can be induced by IFN-α, IFN-γ, and double-stranded RNA. Although IRF1 expression is at basal level in resting cells, it maintains the constitutive, low level of ISG expression, either alone or in combination with STAT1 [173,174]. The low, basal expression of ISGs may be essential for trapid activation of these IFN-activated signaling pathways. HSV-1 microRNA MiR-23a binds to the 3’UTR of IRF1 mRNA and inhibits its expression, contributing to effective HSV-1 replication [175].

## 3. Future Perspectives

Recent studies have significantly advanced our understandings of the biology and particular functions of ISGs in microbial infection. Most of these studies entail various model viruses to examine the role of ISGs in cultured cells, validating an effective approach to addressing this outstanding question and providing a framework for future work. Among the few hundreds ISGs that likely play redundant and distinct roles in host defense against microbial infection, only a small subset of them have been investigated and defined by adequate experiments. It is important to explore new approaches that can systematically determine the role of ISGs in host defense, in addition to the ectopic expression approach [147]. Moreover, it is important to assess their roles in vivo using animal models where genetically modified animal models (such as rodent and zebra fish) can be readily achieved with new technologies (e.g., CRISPR/Cas9). Tissue-specific deletion or expression of ISGs in model animals will offer new insight into the tissue- or organ-specific functions of ISGs, and the coordination thereof, toward building an integrated view on ISG’s network on an organismal level. This will be particularly insightful for viruses that are capable of infecting and causing diseases at multiple anatomical sites. Application of animal models for important human viruses are limited to those that demonstrate infectivity in corresponding animals, and caution has to be exercised in extrapolating the implicated biology on humans.

IFN-independent ISGs often have direct and indirect antiviral activity against viral pathogens. These aspects have not been sufficiently studied for most ISGs, and how these two activities are organized to mesh an effective antiviral immune defense remains an interesting topic to explore. Genetic, biochemical and other approaches are in need to dissect the molecular action of ISGs in viral infection ex vivo and in vivo. Furthermore, ISGs often target more than one step of viral infection, which is very true for herpesviruses that involve diverse cellular processes for their productive infection, necessitating the effort to detail the molecular mechanism of ISG’s action in viral infection. Further compounding this issue is the communication between different tissues and cells involved in viral infection, when the roles of ISGs are examined at the organismal level. Combining cultured cells and animal models will render more detail concerning the action of ISGs in antiviral host defense. Additionally, the basal and IFN-induced expression of ISGs may make distinct contributions to the collective function of ISGs in viral infection, and little has been explored, calling for further investigation. On the other hand, viruses inevitably evolve countermeasures to disarm host defensive mechanisms. Characterizing these viral strategies will instruct us on how cellular defense is regulated, and on new ways to defeat viral infection.
